# Anatomical landmarks for safely implementing resuscitative balloon occlusion of the aorta (REBOA) in zone 1 without fluoroscopy

**DOI:** 10.1186/s13049-017-0411-z

**Published:** 2017-07-03

**Authors:** Yohei Okada, Hiromichi Narumiya, Wataru Ishi, Ryoji Iiduka

**Affiliations:** Department of Emergency and Critical Care Medicine, Japanese Red Cross Society Kyoto Daini Red Cross Hospital, 355-5 Haruobicho Kamigyoku, Kyoto, 602-8026 Japan

**Keywords:** Aortic balloon occlusion (ABO), Resuscitative balloon occlusion of the aorta (REBOA), Hemorrhagic shock, External landmark, Trauma resuscitation

## Abstract

**Background:**

Resuscitative balloon occlusion of the aorta (REBOA) can maintain hemodynamic stability during hemorrhagic shock after a following torso injury, although inappropriate balloon placement may induce brain or visceral organ ischemia. External anatomical landmarks [the suprasternal notch (SSN) and xiphoid process (Xi)] are empirically used to implement REBOA in zone 1. We aimed to confirm if these landmarks were useful for determining a balloon catheter length for safe implementation of REBOA in zone 1 without using fluoroscopy.

**Method:**

We selected 25 successive adult blunt trauma cases requiring contrast-enhanced chest/abdominal computed tomography (CT) treated at our emergency department (in an urban area of Kyoto city, Japan) between October 1, 2016 and January 31, 2017. We retrospectively evaluated anonymized CT images. We used three-dimensional multiplanar reconstructions to measure the length along the aorta’s central axis, from the bilateral common femoral arteries (FA) to the celiac trunk (CeT) (FA–CeT) and to the origin of the left subclavian artery (LSCA) (FA–LSCA). Volume-rendering reconstruction images were used to measure the external distance from common FAs to SSN (FA–SSN) and to Xi (FA–Xi).

**Result:**

FA–LSCA was significantly longer than FA–SSN. FA–CeT was significantly shorter than FA–Xi.

**Discussion:**

Based on these results, the REBOA balloon catheter should be shorter than FA–SSN, and longer than FA–Xi to avoid placement outside zone 1. The advantages of this method are that it can rapidly and easily predict a safe balloon catheter length, and it reflects each patient’s individual torso height.

**Conclusion:**

To safely implement REBOA, the balloon catheter length should be shorter than FA–SSN and longer than FA–Xi. We believe that these anatomical landmarks are good references for safe implementation of REBOA in zone 1 without radiographic guidance.

## Background

Resuscitative balloon occlusion of the aorta (REBOA) in zone 1 [between the left subclavian artery (LSCA) and the celiac trunk (CeT)] is useful for maintaining hemodynamic stability during hemorrhagic shock following a torso injury [1]. This procedure can be simply and rapidly implemented in the emergency department and is a suitable alternative to open aortic cross-clamping [1]. However, REBOA can cause lower limb ischemia [2]. Further, inappropriate balloon placement above zone 1 may induce brain ischemia, and misplacement in front of CeT may cause total occlusion of the celiac arterial flow and severe visceral organ ischemia [1]. It is preferable to perform REBOA using fluoroscopy, although this can delay definitive surgery. Ongoing research seeks a safe method to implement REBOA in zone 1 without using fluoroscopy [3–5]. However, most of these studies focused on male combatants (not representative of the general population) or required additional information, such as body mass index (BMI), cardio vascular risk factor (smoking and diabetes mellitus), and images from contrast-enhanced ultrasonography, which are rarely available in acute settings. Pezy et al. [6] reported a fixed-distance model that defines zone 1 as 414–474 mm from the upper border of the symphysis pubis in a French civilian population, although it seems to be difficult to implement the balloon within this narrow range in emergency settings and may depend on the target population. Thus, a simple and patient-based method is still needed to guide REBOA implementation in zone 1 and facilitate tis safe use in the emergency department or pre-hospital settings. A cadaver study [7] has indicated that mid-sternum (the mid point between the xiphoid process and sternal notch) may be useful for implementing REBOA. However, these findings may not extrapolate well to trauma patients because cadavers are typically representative of older patients and may also exhibit precise morphological differences from living humans [7]. In Japan, some acute care surgeons use similar methods involving SSN and Xi for implementing REBOA in zone 1, although there is no clear evidence to support this approach. We sought to determine if we could use these anatomical landmarks to safely implement REBOA in zone 1 without using fluoroscopy.

## Methods

### Study population

We selected 25 successive adult blunt trauma cases requiring contrast-enhanced chest/abdominal computed tomography (CT) at our emergency department (in an urban area of Kyoto city, Japan) between October 1, 2016 and January 31, 2017. We retrospectively evaluated anonymized CT images. Because this study was a retrospective anonymized review and no intervention was performed, the ethics committee of our hospital stated that this study did not require ethical approval.

**Fig. 1:**
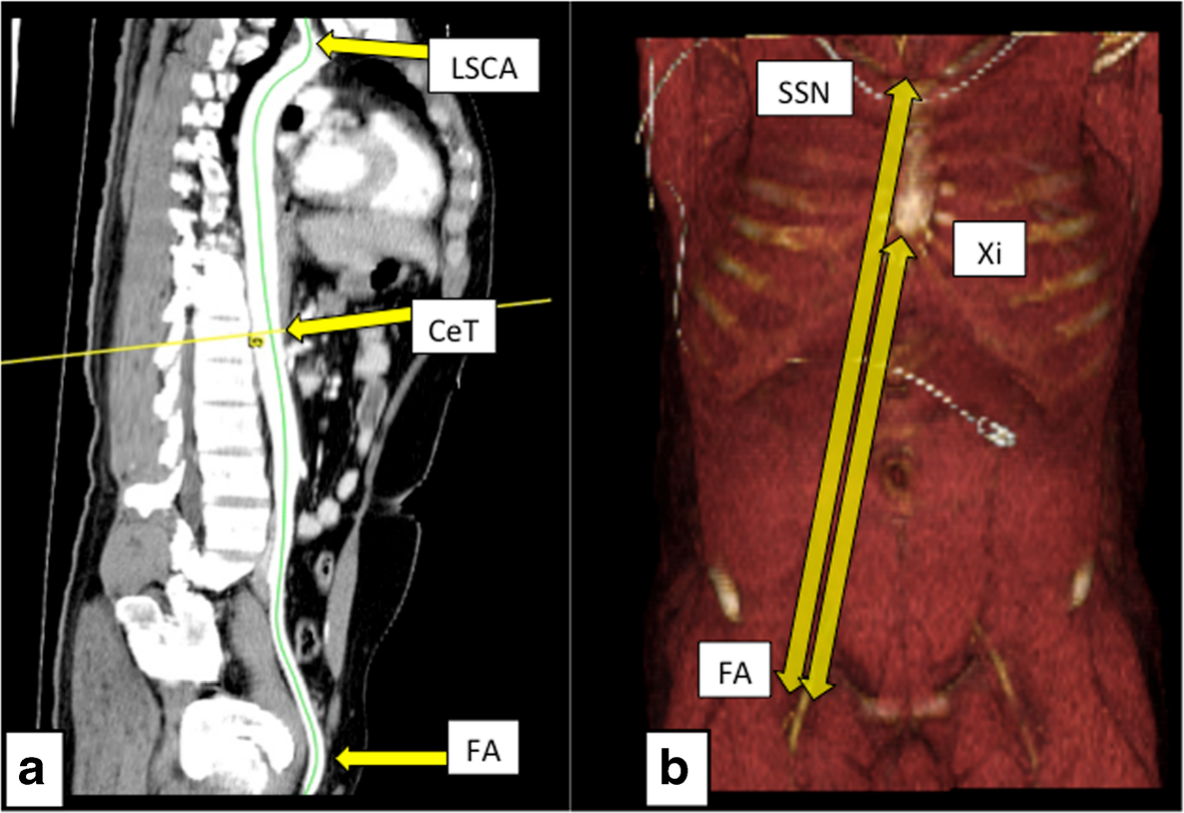
Three-dimensional and volume-rendering images. **a** Three-dimensional multiplanar reconstructions were used to measure the length from the bilateral common femoral artery (FA) to the origin of the left subclavian artery (LSCA) and to the celiac trunk (CeT). **b** Volume-rendering images were used to measure the external distances from the common FAs to the suprasternal notch (SSN) and to the xiphoid process (Xi)

All image analyses were performed using an image processing application for the Mac operating system (Osirix; Pixmeo). Three-dimensional multiplanar reconstructions were used to measure the length along the central aorta axis, from the bilateral common femoral arteries (FA) to the origin of CeT (FA–CeT) and to the origin of LSCA (FA–LSCA) (Fig. 1a). We also measured the external distances from the common FA to SSN (FA–SSN) and to Xi (FA–Xi) using volume-rendering reconstruction images (Fig. 1b). The point of reference for FAs was defined as the common femoral artery in front of the middle of the femoral head, which is the most superficial part of the common femoral artery and is commonly used for arterial access.

All data were compiled on an Excel spreadsheet (Microsoft) and were analyzed using Statcel software (4th edition, Japan). We calculated mean, minimum, and maximum values for patient age, height, and BMI. We also calculated the mean, minimum, 25th percentile, median, 75th percentile, and maximum values for all length measurements. Bilateral FA–LSCA, FA–SSN, FA–CeT, and FA–Xi were compared using a paired *t*-test, and differences with a *p*-value of <0.01 were considered statistically significant.

## Results

Patient data are summarized in Table 1. The mean age was 50 years (range: 21–83 years), and 72% of the patients were male (18/25). Mean values for BMI and height were 22.9 kg/m^2^ (range: 15.5–27.8 kg/m^2^) and 164 cm (range: 145–177 cm), respectively. We only identified one case of traumatic aortic dissection and did not detect any other arterial diseases (e.g., aortic aneurysm). The mean length was 56.2 mm [interquartile range (IQR): 54.1–58.1 mm] for right FA–LSCA, 56.5 mm (IQR: 53.9–58.6 mm) for left FA–LSCA, 53.1 mm (IQR: 51.8–55.0 mm) for right FA–SSN, and 53.4 mm (IQR: 52.0–55.3 mm) for left FA–SSN. The mean length was 38.5 mm (IQR: 37.4–40.4 mm) for right FA–Xi, 38.8 mm (IQR: 38.2–40.1 mm) for left FA–Xi, 31.7 mm (IQR: 30.3–32.9 mm) for right FA–CeT, and 32.0 mm (IQR: 30.5–33.3 mm) for left FA–CeT. All lengths were normally distributed (*χ* ^2^ test, *p* < 0.05) (Table 2).

**Table 1:** Characteristic of the study population

Characteristic	Mean (SD)	Minimum	Median	Maximum
Men (18/25)				
Age (y)	49.5 (21.9)	21	47	83
Height (cm)	167 (4.44)	157	168	177
BMI (kg/m^2^)	22.4 (2.26)	15.5	22.9	25.3
Women (7/25)				
Age (y)	51.3 (18.9)	25	44	80
Height (cm)	157 (7.46)	145	157	167
BMI (kg/m^2^)	24.1 (2.22)	21.4	23.5	27.8
Total (25)				
Age (y)	50 (20.7)	21	47	83
Height (cm)	164 (7.12)	145	165	177
BMI (kg/m^2^)	22.9 (2.33)	15.5	23.0	27.8

*BMI* body mass index

**Table 2:** Measurement of the each length from the common femoral artery

Distribution	Mean (SD)	Minimum	Median	Maximum	*P* value^*^
Right					
FA-LSCA	56.2 (2.46)	52.1	55.8	60.5	0.45
FA-SSN	53.1 (2.51)	46.6	53.4	57.1	0.592
FA-Xi	38.5 (2.26)	33.1	38.3	42.3	0.47
FA-CeT	31.7 (1.47)	30	31.4	35.2	0.357
Left					
FA-LSCA	56.5 (2.84)	52.4	56.8	61.8	0.078
FA-SSN	53.4 (2.49)	47.3	53.8	56.9	0.27
FA-Xi	38.8 (2.42)	32.5	38.8	43	0.27
FA-CeT	32.0 (1.62)	29.4	32.3	35.5	0.13

*FA-LSCA* The artery length between common femoral artery and the origin of left subclavian artery, *FA-SSN* The distance from common femoral artery to supra-sternum notch, *FA-Xi* The distance from common femoral artery to Xiphoid, *FA-CeT* The artery length between common femoral artery and celiac trunk

*Each of them is normally distributed (*χ* ^2^ test, *p* < 0.05)

**Fig. 2:**
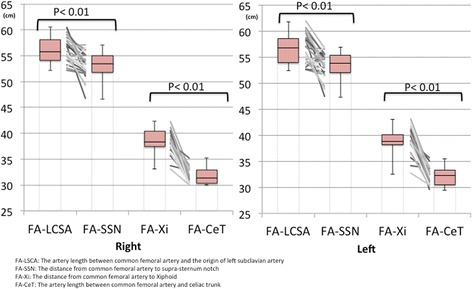
Distributions of the measurements from this study. The box plots indicate that FA–LSCA was significantly longer than FA–SSN, and FA–Xi was significantly longer than FA–CeT on both sides (paired one-tailed t-test, *p* < 0.01). FA: femoral artery, FA–LSCA: the artery length between common femoral artery and the origin of left subclavian artery, FA–SSN: the external distance from common femoral artery to supra-sternum notch, FA–Xi: The external distance from common femoral artery to the xiphoid process, FA–CeT: the artery length between common femoral artery and celiac trunk, LSCA: left subclavian artery, SSN: suprasternal notch, Xi: xiphoid process, CeT: celiac trunk

When we compared bilateral FA–LSCA and FA–SSN, we found that FA–LSCA was significantly longer than FA–SSN (paired one-tailed *t*-test, *p* < 0.01) (Fig. 2). Comparing bilateral FA–Xi and FA–CeT, we found that FA–CeT was significantly shorter than FA–Xi (paired one-tailed t-test, *p* < 0.01) (Fig. 2).

## Discussion

**Fig. 3:**
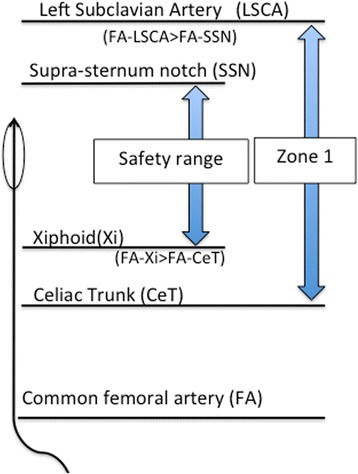
The model for using external anatomical landmarks for predicting a safe balloon catheter length

Our study showed that FA–LCSA was significantly longer than FA–SSN and FA–CeT was significantly shorter than FA–Xi. Based on these findings, the REBOA balloon catheter should be shorter than FA–SSN, and longer than FA–Xi to avoid placement outside zone 1 (Fig. 3). External anatomical landmarks can be useful referents for safely implementation of REBOA in zone 1 without radiographic guidance.

This method for determining the target catheter length has several advantages compared with other previously reported methods. The first advantage is that it can rapidly and easily predict a safe balloon catheter length because it only requires external measurements from the common FA to XI and to SSN. Other methods for placement of REBOA require additional equipment, information, and time. For example, contrast-enhanced ultrasonography is useful for implementing REBOA in zone 3 [3]. Although this approach is relatively non-invasive, the need for contrast medium requires additional equipment. In addition, ultrasonography has limited ability in evaluating the descending aorta, thereby precluding its use in zone 1. Another study has constructed a morphometric map for predicting the distance from the right common FA to the major aortic branches [4], although it involves complex calculations that require additional information, such as the patient’s medical history and BMI. Thus, this approach cannot be simply and rapidly used in chaotic trauma resuscitation settings.

The second advantage of this method is that it accounts for the patient’s torso height. In the fixed-distance model, zone 1 was defined as 414–474 mm from the symphysis pubis in a population of trauma patients from an urban area in France. However, this model was not based on each patient’s individual torso height. Moreover, placement can be difficult in such a narrow range. In contrast, anatomical landmarks reflect each patient’s individual torso height. This method may, therefore, give us a wider safety margin to ensure placement in zone 1. We believe that the external anatomical landmark method is a simple and patient-specific method for safely implementing REBOA in zone 1.

A similar approach was proposed in the previous cadaver study [7]. However, we believe that our study is more suited for application with general trauma victims, in urban areas, comparing with the cadaver study. In the cadaver study, older age [median age: 70 (range: 43–75) years], and smaller BMI (mean ± SDBMI: 19.4 ± 3.1 kg/m^2^) of cadavers limits the ability to translate these results to typical trauma patients [7]. Changes in aortic morphology in the cadavers may further limit to translate these findings to younger patients [7]. It is unknown if the cadaver study findings are representative of younger trauma victims.

We selected actual adult blunt trauma victims from an urban area. Our study population was younger [median age: 47 (range: 21–83) years] with an average BMI (mean ± SDBMI: 22.4 ± 2.26 kg/m^2^) and may, therefore, be more suitable for application in the general trauma victim population. Our results build upon those of the cadaver study to confirm the acceptability of external landmarks for implementation of REBOA in zone 1.

This study had several limitations. First, it is unclear if our population was representative of all trauma patients because we only evaluated Japanese adults. Additional studies with different populations (particularly those with higher BMI and other nationalities) are needed to confirm if our approach is appropriate for all trauma patients. Second, we do not know if our measurements reflect the clinical requirements of living humans because the advancement and placement of the balloon catheter can be affected by the cardiac output.

Lastly, we believe that this method may be useful for safely implementing REBOA in emergency settings, without radiographic guidance. Importantly, our findings do not mean that it is unnecessary to confirm balloon position by other methods, such as portable chest X-ray in the trauma bay. We should always consider anatomical abnormalities and unexpected misplacement, and radiologically confirm the position as soon as possible to maximize patient safety.

## Conclusion

FA–LCSA was significantly longer than FA–SSN, and FA–Xi was significantly longer than FA–CeT. External anatomical landmarks (SSN and Xi) can be used to determine the required balloon catheter length (shorter than FA–SSN and longer than FA–Xi). These anatomical landmarks are useful for safe implementation of REBOA in zone 1 without radiographic guidance.

## Abbreviations

BMI: Body mass index
CeT: Celiac trunk
FA: Femoral artery
FA–CeT: The artery length between common femoral artery and celiac trunk
FA–LSCA: The artery length between common femoral artery and the origin of left subclavian artery
FA–SSN: The external distance from common femoral artery to supra-sternum notch
FA–Xi: The external distance from common femoral artery to the xiphoid process
LSCA: Left subclavian artery
REBOA: Resuscitative balloon occlusion of the aorta
SSN: Suprasternal notch
Xi: Xiphoid process
